# Thyroid function, body mass index, and metabolic risk markers in euthyroid adults: a cohort study

**DOI:** 10.1186/s12902-019-0383-2

**Published:** 2019-06-07

**Authors:** Ranran Xu, Fei Huang, Shijie Zhang, Yongman Lv, Qingquan Liu

**Affiliations:** 1 Department of Nephrology, Tongji Hospital, Tongji Medical College, Huazhong University of Science and Technology, 1095 Jie Fang Avenue, Hankou, Wuhan, 430030 People’s Republic of China; 2 Health Management Center, Tongji Hospital, Tongji Medical College, Huazhong University of Science and Technology, 1095 Jie Fang Avenue, Hankou, Wuhan, 430030 People’s Republic of China

**Keywords:** Body mass index, Obesity, Thyroid-stimulating hormone, Triiodothyronine, Thyroxine, Dyslipidemia

## Abstract

**Background:**

In recent years, the relationship between thyroid stimulating hormone (TSH) and obesity has been widely discussed. However, it is unclear how thyroid hormone concentrations relate to body weight and its impact on metabolic risk markers. This study aimed to assess how thyroid function is linked to underweight, overweight, or obesity, and metabolic risk markers in adults.

**Methods:**

A total of 16,975 subjects, aged 18–80 years, who attended the Health Management Center of Tongji Hospital, Wuhan, China were enrolled in this study. Anthropometric and laboratory data were collected and analyzed.

**Results:**

Serum free triiodothyronine (fT3) and fT3/free thyroxine (fT4) ratio (fT3/fT4) were positively associated with body mass index (BMI) (*P* < 0.001), while there was a negative relationship between fT4 and BMI (*P* < 0.001) according to multivariable regression analysis adjusted for age and sex. Associations between thyroid hormone concentrations and markers of blood pressure, and lipid and glucose metabolism were identified after adjustment for age, sex, and BMI, with TSH being negatively associated with fasting blood glucose (FBG). fT3 was positively associated with systolic blood pressure and low-density lipoprotein-cholesterol, while fT4 was positively associated with diastolic blood pressure, FBG, and high-density lipoprotein-cholesterol (HDL-C), and negatively associated with hemoglobin A1c (HbA1c) and triglyceride. Finally, fT3/fT4 was positively associated with HbA1c and triglyceride, and negatively associated with HDL-C.

**Conclusions:**

Overweight or obese participants had a high serum concentration of fT3, high fT3/fT4 ratio, and a low concentration of fT4. Underweight participants had high concentrations of fT4 and low concentrations of fT3. Thus, relationships between thyroid hormones and metabolic risk markers were identified which suggest that thyroid function might be one factor that influences body weight and the co-morbidities of obesity.

## Background

Thyroid hormones play an important role in regulating thermogenesis, and glucose and lipid metabolism, which make them a key factor regulating mammalian dynamic energy balance [1]. The effects of hypo- or hyperthyroidism on body weight were observed a long time ago. The low thyroid hormone concentrations that characterize hypothyroidism are associated with lower energy expenditure [2] and fluid retention [3], whereas hyperthyroidism is often associated with an increase in energy expenditure and weight loss. However, the relationship between thyroid function within the normal range and body weight has not been fully characterized.

Twenty percent of severely obese people have subclinical hypothyroidism [4], which is defined by the presence of a high concentration of thyroid stimulating hormone (TSH), but a normal concentration of thyroxin (T4) or free thyroxin (fT4) [5]. Some scholars have attempted to attribute obesity to subclinical hypothyroidism [6]. However, studies have shown that in obese patients, in addition to the high TSH concentration, there are also high concentrations of free triiodothyronine (fT3) [7, 8] and fT4 [7], which contrasts with subclinical hypothyroidism, during which serum fT4 and fT3 concentrations are at the lower end of the normal range. This is inconsistent with subclinical hypothyroidism being a cause of obesity, and therefore the relationship between thyroid function and body weight requires further study.

Several studies have evaluated the relationship between obesity and thyroid function in euthyroid adults [9–12]. Muscogiuri et al. [10] reported that obesity is positively associated with TSH concentration, while other researchers have found no association between adiposity and TSH [11, 12], triiodothyronine (T3) [11, 12], or fT4 [11]. In pediatric cohort studies, TSH concentration has been shown to be positively associated with body mass index (BMI) and fat mass [6, 13–15]. Jin et al. [6] reported a negative association between fT4 and BMI, but Lundback et al. [13] did not find any association between adiposity and fT3 or fT4. However, these studies were of relatively small samples, which may explain their contrasting conclusions. Therefore, larger scale investigations of the relationships between TSH secretion, fT3, and fT4 with body weight in the general population are required.

TSH concentrations within the normal range have previously been reported to be associated with metabolic risk factors in obese children [6, 13] and in adults [16]. Jin et al. [6] have shown that serum TSH is associated with lipid profile in obese children and adolescents, while Rahbar et al. [16] found no significant differences between a high-TSH and a low-TSH group with regard to serum triglyceride (TG), total cholesterol (TC), and high-density lipoprotein-cholesterol (HDL-C) concentrations in euthyroid adults. These disparities imply a lack of clarity in the literature with regard to whether TSH and thyroid hormones regulate metabolism in obese patients.

The purpose of this study was to elucidate the relationships between thyroid hormone concentrations within the normal range and body weight, including in overweight, obese, and underweight individuals, and metabolic status in the general population, using a large sample. The findings hence represent a snapshot of normal physiology and no causal relationship can be inferred.

## Methods

### Study participants

**Fig. 1:**
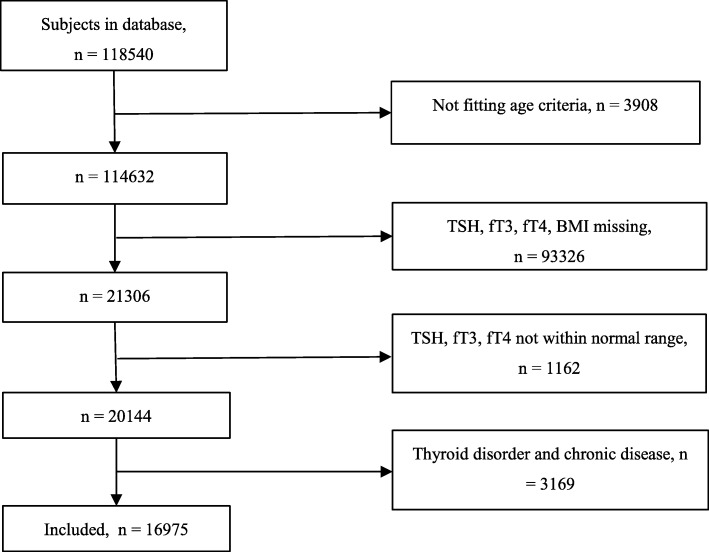
Flow diagram describing the selection strategy. The participants were 18–80 years old. Reference ranges for thyroid-stimulating hormone (TSH): 0.35–4.94 μIU/ml, free triiodothyronine (fT3): 1.71–3.71 pg/ml, free thyroxine (fT4) 0.7–1.48 ng/dl=

This was a cross-sectional study conducted between January 1st and December 31th, 2017, at the Health Management Center of Tongji Hospital, Wuhan, China. All the participants who underwent a health check during 2017 were screened for their eligibility to enroll in this study. They all underwent a routine medical examination, had anthropometric measurements made, and provided overnight fasting blood samples and information regarding their medical history, as part of the health check. We selected subjects 18–80 years, in whom thyroid hormone concentrations had been measured and were within the normal range. Participants who were being treated because of a thyroid disorder, or who were current users of drugs known to influence thyroid function, including lithium, amiodarone, estrogen, and corticosteroids, were excluded. In addition, participants with diabetes, cardiovascular disease, chronic lung disease, cancer, renal failure, autoimmune disease, inflammation, pregnancy, or other diseases that might affect thyroid hormone concentrations or metabolism were excluded. A flow diagram describing the selection strategy is shown as Fig. 1.

After participants had been excluded on the basis of the criteria listed above, a total of 16,975 subjects were enrolled, and they provided written informed consent before participating. The study protocol was approved by the Ethics Committee of Tongji Hospital, Tongji Medical College, Huazhong University of Science and Technology.

### Anthropometric measurements

The height and body mass of the participants were measured using a stadiometer, in the absence of a coat and shoes. BMI was calculated as body mass (kg) divided by the square of height (m^2^), and participants were categorized as underweight (BMI < 18.5 kg/m^2^), normal weight (18.5 kg/m^2^ ≤ BMI < 23.9 kg/m^2^), overweight (24.0 kg/m^2^ ≤ BMI < 27.9 kg/m^2^), or obese (BMI ≥28 kg/m^2^), according to the 2003 Working Group on Obesity in China (WGOC) guidelines [17]. Blood pressure was measured in the sitting position after 10 min of rest using an electronic sphygmomanometer (HBP-9020; Omron, Dalian, China).

### Biochemical measurements

Blood samples were obtained after an overnight fast, and routine biochemical analyses (TSH, fT3, fT4, TC, TG, low-density lipoprotein-cholesterol [LDL-C], and HDL-C) were performed at accredited hospital laboratories. The reference ranges were: TSH 0.35–4.94 μIU/ml, fT3 1.71–3.71 pg/ml, fT4 0.7–1.48 ng/dl, TC < 5.8 mmol/L, TG < 1.70 mmol/L, HDL-C 1.04–1.55 mmol/L, and LDL-C < 3.37 mmol/L. Dyslipidemia was classified as previously [18]: hypercholesterolemia: total cholesterol ≥5.8 mmol/L; hypertriglyceridemia: triglyceride ≥1.70 mmol/L; mixed hyperlipidemia: total cholesterol ≥5.8 mmol/L and triglyceride ≥1.70 mmol/L; low HDL-C: high-density lipoprotein ≤1.04 mmol/L.

### Statistical analysis

Data were processed and analyzed using IBM SPSS software version 22.0 (IBM Inc., Armonk, NY, USA). Continuous variables are expressed as mean ± standard deviation and were compared using Student’s *t* test. The relationships between thyroid hormone concentrations and other parameters were evaluated using Pearson’s correlation. Multiple linear regressions were used to assess the relationships between thyroid parameters and metabolic risk markers. Odds ratios (ORs) and 95% confidence intervals (CIs) for quartiles of thyroid parameters obtained from logistic regression models were used to predict the risk of overweight, obesity, and dyslipidemia, after adjustment for age and sex. All statistical tests were two-sided and *P* < 0.05 was considered to indicate statistical significance.

## Results

### Characteristics of the participants

A total of 16,975 euthyroid individuals of the 118,540 people attending the Health Management Center of Tongji Hospital were included in this study. Their mean age was 41.7 ± 10.5 years and 9523 (56.1%) were men. The participants were classified into four groups (underweight, normal weight, overweight, or obese). The baseline characteristics of the participants in each group are presented in Table 1. Independent Student’s *t*-tests showed there were significant differences between the normal weight group and other weight groups in most anthropometric and laboratory indexes. Of these, systolic blood pressure (SBP), diastolic blood pressure (DBP), fT3, fT3/fT4 ratio, fasting blood glucose (FBG), hemoglobin A1c (HbA1c), TG, TC, LDL-C, and homocysteine were higher in the higher weight groups, whereas fT4 and HDL-C were higher in the lower weight groups (Table 1).

**Table 1:** Characteristics of the study population

Characteristics	Low weight	Normal weight	Overweight	Obese
Number (Male/female)	737 (168/569)	8671 (3636/5035)	5943 (4404/1539)	1624 (1315/309)
Age, years	34 ± 11**	41 ± 11	44 ± 10**	43 ± 10**
Height, cm	163.3 ± 7.1**	164.7 ± 7.8	167.7 ± 7.9**	168.5 ± 7.6**
Weight, kg	47.0 ± 4.6**	58.7 ± 7.3	72.2 ± 7.6**	85.4 ± 9.9**
BMI, kg/m^2^	17.6 ± 0.7**	21.6 ± 1.4	25.6 ± 1.1**	30.0 ± 2.1**
SBP, mmHg	110 ± 12**	116 ± 15	125 ± 16**	134 ± 17**
DBP, mmHg	68 ± 9**	71 ± 11	78 ± 11**	84 ± 12**
TSH, uIU/ml	1.89 ± 0.87	1.88 ± 0.89	1.84 ± 0.86*	1.90 ± 0.90
fT3, pg/ml	2.80 ± 0.28*	2.83 ± 0.29	2.91 ± 0.29**	2.97 ± 0.29**
fT4, ng/dl	1.05 ± 0.10**	1.03 ± 0.11	1.01 ± 0.11**	1.01 ± 0.11**
fT3/ fT4	2.68 ± 0.32**	2.78 ± 0.37	2.91 ± 0.39**	2.96 ± 0.40**
FBG, mmol/l	4.92 ± 0.59**	5.08 ± 0.68	5.32 ± 0.95**	5.63 ± 1.28**
HbA1c, %	5.31 ± 0.25**	5.44 ± 0.47	5.58 ± 0.55**	5.77 ± 0.78**
TG, mmol/l	0.80 ± 0.30**	1.15 ± 0.83	1.81 ± 1.48**	2.32 ± 2.32**
TC, mmol/l	4.19 ± 0.76**	4.46 ± 0.83	4.69 ± 0.87**	4.81 ± 0.90**
LDL-C, mmol/l	2.31 ± 0.62**	2.65 ± 0.70	2.87 ± 0.74**	2.95 ± 0.79**
HDL-C, mmol/l	1.58 ± 0.30**	1.37 ± 0.30	1.16 ± 0.25**	1.07 ± 0.21**
Homocysteine, umol/l	11.71 ± 5.79	11.91 ± 6.65	12.76 ± 6.36**	13.66 ± 7.74**

*BMI* body mass index, *SBP* systolic blood pressure, *DBP* diastolic blood pressure, *TSH* thyroid stimulating hormone, *fT3* free triiodothyronine, *fT4* free thyroxine, *FBG* fasting blood glucose, *HbA1c* hemoglobin A1c, *TC* total cholesterol, *TG* triglyceride, *LDL-C* low-density lipoprotein-cholesterol, *HDL-C* high-density lipoprotein-cholesterol. Underweight, BMI < 18.5 kg/m^2^; Normal weight, 18.5 kg/m^2^ ≤ BMI < 23.9 kg/m^2^; Overweight, 24.0 kg/m^2^ ≤ BMI < 27.9 kg/m^2^; Obese, BMI ≥ 28 kg/m^2^. Data are expressed as means ± SD. * *p* < 0.05 versus Normal weight; ** *p* < 0.001 versus Normal weight

### Relationships between thyroid parameters and metabolic indexes

According to Pearson correlation analysis, thyroid function indicators were not linearly correlated with most metabolic risk factors, given that these significant *p* values do not make the correlations clinically significant. Multivariable regression analysis, adjusted for age and sex, showed no significant correlations between TSH and BMI, but fT3 and fT3/fT4 were positively correlated with BMI, and fT4 was negatively correlated with BMI (all *P <* 0.001) (Table 2). Higher SBP was associated with higher fT3 concentration (*P* = 0.004) and DBP was positively correlated with fT4 (*P* < 0.001). FBG was negatively associated with TSH (*P* = 0.006) and positively associated with fT4 (*P* < 0.001). Higher HbA1c concentration was associated with lower fT4 (*P* = 0.017) and higher fT3/fT4 (*P* = 0.002). Serum TG was positively associated with TSH and fT3/fT4, and negatively associated with fT4 (all *P* < 0.001). No significant association was observed between TC and TSH, fT3, fT4 or fT3/fT4. Higher LDL-C concentration was only associated with a higher concentration of fT3 (*P* = 0.009), and higher HDL-C was only associated with higher fT4 (*P* = 0.005). There was no relationship between serum homocysteine and TSH, fT3, fT4, or fT3/fT4.

**Table 2:** Associations of thyroid function with components of the metabolic syndrome

	TSH				fT3				fT4				fT3/ fT4			
	r	P _r_	β	P_β_	r	P _r_	β	P_β_	r	P _r_	β	P_β_	r	P _r_	β	P_β_
BMI	−0.004	0.574	0.012**	< 0.001	0.192**	< 0.001	0.008**	< 0.001	−0.073**	< 0.001	−0.003**	< 0.001	0.209**	< 0.001	0.016**	< 0.001
SBP	0.011	0.161	0.014	0.49	0.151**	< 0.001	0.001**	0.004	0.048**	< 0.001	0.035	0.271	0.082**	< 0.001	−0.02	0.302
DBP	0.004	0.624	0.032	0.102	0.156**	< 0.001	0.006	0.84	0.040**	< 0.001	0.001**	< 0.001	0.094**	< 0.001	−0.031	0.112
FBG	−.0028**	< 0.001	−0.051**	0.006	0.046**	< 0.001	−0.006	0.728	0.041**	< 0.001	0.012**	< 0.001	0.009	0.22	−0.042**	< 0.001
HbA1c	−0.013	0.219	0.001	0.988	0.012	0.242	0.01	0.571	−0.027**	0.008	−0.013*	0.017	0.036**	0.001	0.055**	0.002
TG	0.006	0.458	0.042**	< 0.001	0.120**	< 0.001	0.014	0.443	−0.080**	< 0.001	−0.005**	< 0.001	0.162**	< 0.001	0.015**	< 0.001
TC	0.030**	< 0.001	0.008	0.661	0.025**	0.001	−0.01	0.729	− 0.002	0.801	0.034	0.105	0.027**	0.001	0.004	0.189
LDL-C	0.005	0.538	0.003	0.878	0.068**	< 0.001	0.018**	0.009	0.025**	0.001	0.033	0.074	0.034**	< 0.001	0.009	0.608
HDL-C	0.045**	< 0.001	0.021	0.323	−0.199**	< 0.001	−0.014	0.468	0.061**	< 0.001	0.023**	0.005	−0.201**	< 0.001	−0.065*	0.018
Homocysteine	−0.053**	0.002	−0.001	0.957	0.039*	0.021	−0.003**	< 0.001	0.038*	0.025	0.033	0.077	0.001	0.992	−0.005**	< 0.001

Values of r represent correlation coefficient. Values of β are standardized regression coefficients. P _r_ represents the significance for r. P_β_ represents the significance for β. BMI was estimated after adjustment for gender and age. Other variables were estimated after adjustment for gender, age and BMI. *p < 0.05; ** *p* < 0.01

*BMI* body mass index, *SBP* systolic blood pressure, *DBP* diastolic blood pressure, *TSH* thyroid stimulating hormone, *fT3* free triiodothyronine, *fT4* free thyroxine, *FBG* fasting blood glucose, *HbA1c* hemoglobin A1c, *TC* total cholesterol, *TG* triglyceride, *LDL-C* low-density lipoprotein cholesterol, *HDL-C* high-density lipoprotein cholesterol

### Risks of underweight, overweight, obesity, or dyslipidemia

According to logistic regression analyses, quartiles 2 to 4 of fT4 serum concentration were associated with a higher risk of underweight than quartile 1 (ORs for quartiles 2–4 were 2.526, 2.499, and 1.770, respectively), after adjustment for age and sex. The higher quartiles of TSH (quartiles 2–3), fT3 (quartiles 3–4) and fT4 (quartiles 2–4) concentration were associated with a higher risk of overweight. In addition, the higher quartiles of TSH (quartiles 2–4) and fT3/fT4 (quartiles 2–4) were associated with a higher risk of obesity. ORs of 1.71 and 4.14 were obtained for the highest TSH and fT3/fT4 quartiles, respectively, when they were compared with the lowest quartiles for the prediction of obesity (Table 3).

**Table 3:** Logistic regression analyses of the risk of underweight, overweight, and obesity

	Quartile	Low weight	Overweight	Obese
TSH	1	1	1	1
	2	1.150(0.970–1.363)	1.102(1.020–1.191) #	1.214(1.075–1.372) #
	3	1.097(0.860–1.401)	1.123(1.004–1.257) #	1.359(1.141–1.618) #
	4	1.021(0.675–1.544)	1.063(0.881–1.283)	1.714(1.307–2.249) #
fT3	1	1	1	1
	2	1.159(0.611–2.196)	1.227(0.886–1.698)	0.947(0.513–1.751)
	3	1.134(0.586–2.195)	1.437(1.029–2.007) #	1.217(0.652–2.270)
	4	0.855(0.406–1.802)	1.525(1.065–2.183) #	1.500(0.782–2.878)
fT4	1	1	1	1
	2	2.526(1.269–5.028) #	0.815(0.721–0.922) #	0.993(0.819–1.205)
	3	2.499(1.656–3.770) #	0.703(0.601–0.821) #	0.944(0.738–1.209)
	4	1.770(1.223–2.562) #	0.487(0.345–0.687) #	0.941(0.557–1.588)
fT3/ fT4	1	1	1	1
	2	0.954(0.698–1.303)	1.020(0.854–1.218)	1.460(1.045–2.040) #
	3	0.873(0.552–1.381)	1.265(1.015–1.578) #	2.287(1.550–3.374) #
	4	0.763(0.654–1.271)	1.088(0.525–2.253)	4.140(1.727–9.925) #

*TSH* thyroid stimulating hormone, *fT3* free triiodothyronine, *fT4* free thyroxine. Low weight, BMI < 18.5 kg/m^2^; Normal weight, 18.5 kg/m^2^ ≤ BMI < 23.9 kg/m^2^; Overweight, 24.0 kg/m^2^ ≤ BMI < 27.9 kg/m^2^; Obese, BMI ≥ 28 kg/m2. The cut-offs for the quartiles of TSH were 1.226 μIU/ml, 1.687 μIU/ml, and 2.333 μIU/ml; for fT3 2.68 pg/ml, 2.87 pg/ml, and 3.07 pg/ml; for fT4 0.94 ng/dl, 1.01 ng/dl, and 1.09 ng/dl; and for fT3/fT4 2.58, 2.83, and 3.09

OR and 95% CI were calculated using logistic regression models and adjusted for age and sex. # *p* < 0.05

Table 4 shows the thyroid parameters, divided into quartiles, in the presence of dyslipidemia. Dyslipidemia was classified as hypercholesterolemia, hypertriglyceridemia, mixed hyperlipidemia, or low HDL-C concentration, on the basis of the Chinese Guidelines on the Prevention and Treatment of Dyslipidemia in Adults [18]. The highest TSH quartile was associated with a higher risk of hypercholesterolemia (OR, 1.39; 95% CI, 1.07–1.81), hypertriglyceridemia (OR, 1.35; 95% CI, 1.11–1.63), and mixed hyperlipidemia (OR, 1.68; 95% CI, 1.19–2.36). The highest fT3 quartile predicted hypertriglyceridemia with an OR of 1.64 (95% CI, 1.10–2.46), the lower quartiles of fT4 were associated with a higher risk of hypertriglyceridemia and low HDL-C, and the higher quartiles of fT3/fT4 were associated with a higher risk of low HDL-C.

**Table 4:** Logistic regression analyses of the risk of dyslipidemia

	Quartile	Hypercholesterolemia	Hypertriglyceridemia	Mixed hyperlipidemia	Low HDL-C
TSH	1	1	1	1	1
	2	1.043(0.918–1.184)	1.163(1.073–1.260) #	0.967(0.816–1.145)	1.074(0.989–1.167)
	3	1.037(0.864–1.243)	1.192 (1.060–1.340) #	1.059(0.830–1.351)	1.118(0.989–1.265)
	4	1.391(1.066–1.814) #	1.349(1.114–1.634) #	1.678(1.193–2.360) #	1.215(0.986–1.497)
fT3	1	1	1	1	1
	2	1.169(0.712–1.920)	1.230(0.847–1.787)	1.128(0.547–2.325)	1.190(0.772–1.832)
	3	1.123(0.672–1.874)	1.412(0.964–2.068)	1.111(0.527–2.339)	1.279(0.824–1.984)
	4	1.277(0.735–2.219)	1.643(1.098–2.459) #	1.276(0.580–2.810)	1.325(0.838–2.096)
fT4	1	1	1	1	1
	2	1.205(0.982–1.478)	0.736(0.651–0.833) #	1.085(0.837–1.407)	0.858(0.752–0.979) #
	3	1.268 (0.981–1.640)	0.615(0.524–0.722) #	0.989(0.704–1.389)	0.772(0.653–0.914) #
	4	1.567(0.955–2.572)	0.414(0.284–0.604) #	0.648(0.287–1.465)	0.598(0.408–0.877) #
fT3/ fT4	1	1	1	1	1
	2	0.829(0.636–1.081)	0.956(0.784–1.166)	0.753(0.512–1.107)	1.320(1.061–1.642) #
	3	0.983(0.701–1.379)	1.244(0.981–1.579)	1.000(0.624–1.602)	1.442(1.114–1.867) #
	4	0.703(0.203–2.433)	1.855(0.976–3.525)	0.290(0.037–2.280)	2.262(1.184–4.320) #

*TSH* thyroid stimulating hormone, *fT3* free triiodothyronine, *fT4* free thyroxine, *HDL-C* high-density lipoprotein cholesterol. Hypercholesterolemia, total cholesterol ≥5.8 mmol/L; hypertriglyceridemia, triglyceride ≥1.70 mmol/L; mixed hyperlipidemia, total cholesterol ≥5.8 mmol/L and triglyceride ≥1.70 mmol/L; low HDL-C, high-density lipoprotein ≤1.04 mmol/L. The cut-offs for the quartiles of TSH were 1.226 μIU/ml, 1.687 μIU/ml, and 2.333 μIU/ml; for fT3 2.68 pg/ml, 2.87 pg/ml, and 3.07 pg/ml; for fT4 0.94 ng/dl, 1.01 ng/dl, and 1.09 ng/dl; and for fT3/fT4 2.58, 2.83, and 3.09

OR and 95% CI were calculated using logistic regression models and adjusted for age and sex. # *p* < 0.05

## Discussion

It is well known that thyroid hormones regulate heat production; indeed about 30% of the heat generated to maintain body temperature is dependent on thyroid hormone action [19]. The relationship between body weight and the hypothalamic-pituitary-thyroid axis (HPT axis) is intriguing; for example, some researchers have found that small changes in thyroid function can cause significant changes in weight in overweight patients, who are often thought to be obese due to thyroid dysfunction [20, 21].

Several studies have investigated the relationship between obesity and TSH in euthyroid children [6, 13], finding that BMI is positively correlated with the serum concentration of TSH and negatively correlated with the serum concentration of fT4 after adjusting for age [6], or conversely no significant associations between fT3 or fT4 and BMI [13]. In addition, other pediatric studies found no association between fT4 and BMI [21–23]. Because of these contradictory findings, we undertook the present study using a large sample of 16,975 patients as an adjunct to a comprehensive health examination using standard procedures. To better understanding the specific role of thyroid function in weight regulation, we compared indexes of thyroid function in underweight, normal, overweight and obese participants.

In the present study, a high concentration of fT3 and a low concentration of fT4 were associated with a high risk of overweight. Furthermore, a high concentration of TSH and fT3/fT4 were associated with a higher risk of obesity, implying that the differences in the HPT axis in obesity do not purely represent an exacerbation of the differences present in overweight. In the underweight group, serum TSH concentration was similar to that of normal weight participants, but fT3 concentration and fT3/fT4 were lower, and fT4 was higher. In addition, patients in the first quartile of fT4 had a lower risk of being underweight than the other participants.

Leptin has been reported to be a key component of the complex physiologic regulation of the HPT axis [24] and serum leptin is associated with TSH concentration [25]. In obesity, the leptin concentration increases, and it crosses the blood-brain barrier to regulate the endocrine system through actions in the arcuate nucleus of the hypothalamus [26]. Leptin signaling has also been shown to be required for the maintenance of thyrotropin-releasing hormone (TRH) expression in the hypothalamic paraventricular nucleus [27]. Several epidemiologic studies have found a positive relationship between serum TSH and leptin concentrations in humans [25]. However, conversely, TSH receptors located on adipose cell membranes regulate adipocyte proliferation [28], which may partly explain the association between high TSH concentration and a higher risk of overweight or obesity. Greater secretion of TRH and TSH from the hypothalamus and pituitary leads to the secretion of more thyroid hormone, which would have a compensatory effect to increase metabolism and energy consumption in the periphery. This increase in resting energy expenditure may represent an adaptive response to weight gain.

In addition, TSH increases deiodinase activity and therefore the preferential production of T3 in thyroid cell cultures [29, 30]. Recent studies have shown that at least within the normal range of thyroid function, TSH preferentially increases serum fT3 over fT4 [31], which may at least partly explain the higher fT3 and fT3/fT4 alongside the higher TSH in the overweight and obese groups, in the absence of a similar difference in fT4.

Analysis of the relationships between underweight, and TSH and thyroid hormone concentrations showed no difference in the hypothalamic-pituitary axis (H-P axis) from the normal weight group, while thyroid hormone concentrations showed the opposite difference to that of the overweight or obese groups. TSH and fT3 showed a dependent change in the overweight or obese group, but not in the underweight group, implying that the differences in HPT axis activation in underweight are not just the reverse of those identified in overweight or obesity. However, the mechanism of the relationship between underweight and thyroid function requires further exploration.

It is well known that thyroid hormones can affect serum cholesterol concentration [32]. Here, we investigated the relationships between TSH and thyroid hormones, and serum lipid concentrations, and found that individuals in the higher quartiles of TSH concentration have higher risks of hypercholesterolemia, hypertriglyceridemia, and mixed hyperlipidemia, independent of the effect of BMI. fT3 concentration was positively linked with LDL-C concentration, the lower quartiles of fT4 were associated with hypertriglyceridemia and low serum HDL-C, and high fT3/fT4 was associated with a higher risk of low HDL-C concentration. Thus, TSH and thyroid hormones, even within the normal range of serum concentrations, can influence lipid metabolism. This reflects the sensitivity of blood lipids to minor changes in the activity of the HPT axis, but more research should be conducted to investigate the mechanism underlying the link between blood lipid and thyroid hormones.

Thyroid function is also associated with other metabolic/cardiovascular parameters, such as blood pressure and glucose metabolism. The results showed that SBP was positively correlated with fT3, and DBP was positively correlated with fT4. Previous study by Roos et al. [33] found a correlation between fT4 level and blood pressure and suggested that serum fT3 and fT4 are risk factors for cardiovascular disease rather than serum TSH. The effect of thyroid function on blood pressure is complex and the mechanism is not well understood. It is well known that thyroid diseases can affect cardiac output, peripheral vascular resistance, renal hemodynamics, sodium homeostasis, vascular endothelial function, renin-angiotensin-aldosterone system and many other aspects. This study also found that FBG was negatively correlated with TSH and positively correlated with fT4, and high HbA1c was associated with low serum fT4 and high fT3/fT4. Thyroid hormones are important determinants of glucose homeostasis. Relevant studies have shown that hypothyroidism and subclinical hypothyroidism can cause insulin resistance, and even high TSH in the normal range is positively correlated with insulin resistance [34, 35].

The relationship between thyroid function and non-alcoholic fatty liver disease (NAFLD) has received extensive attention recently [36–38]. It has been reported that in the euthyroid population, fT3 and TSH concentrations are positively associated with the risk of NAFLD [37], and Bril et al. found that low plasma fT4 is associated with a high prevalence of NAFLD [36]. Although we did not have access to data with which to fully assess the degree of risk of NAFLD, we recommend that physicians pay attention to thyroid function in overweight or obese people to prevent the adverse effects of dysregulation of the HPT on metabolism and the liver.

The present study had a number of strengths and weaknesses. We studied a large dataset, in which all the participants had undergone standardized anthropometric and biochemical measurements, thereby minimizing the effects of measurement error. However, we are unable to infer causality, because this was a retrospective cross-sectional study. Furthermore, we were unable to account for a number of confounding factors, including smoking, alcohol, diet, and physical activity, information regarding which was not recorded at the health checks.

## Conclusions

Compared with normal weight individuals, overweight individuals are more likely to have high serum concentrations of fT3 and low concentrations of fT4; obese individuals are more likely to have high concentrations of TSH and fT3/fT4; and underweight individuals are more likely to have high concentrations of fT4 and low concentrations of fT3. Relationships between thyroid parameters and metabolic risk factors were also observed, which suggest that thyroid function is one factor that influences body weight and the development of co-morbidities of obesity. Further longitudinal studies are required to determine whether these are causal relationships, and more research is needed to determine whether interventions should be recommended to reduce the unfavorable effects of HPT axis dysregulation in obesity.

## Data Availability

The data that support the findings of this study are available on request from the corresponding author Yongman Lv. The data have not been made publicly available because they contain information that could compromise the privacy or consent of the study participants.

## Abbreviations

BMI: Body mass index
BP: Blood pressure
FBG: Fasting blood glucose
fT3: free triiodothyronine
fT3/ fT4: free triiodothyronine/free thyroxine ratio
fT4: free thyroxine
HbA1c: Hemoglobin A1
HDL-C: High-density lipoprotein cholesterol
HPT axis: Hypothalamic-pituitary-thyroid axis
LDL-C: Low-density lipoprotein cholesterol
NAFLD: Non-alcoholic fatty liver disease
TC: Total cholesterol
TG: Triglyceride
TRH: Thyrotropin-releasing hormone
TSH: Thyroid stimulating hormone
